# Women with family cancer history are at risk for poorer physical quality of life and lower self-efficacy: a longitudinal study among men and women with non-small cell lung cancer

**DOI:** 10.1186/s12955-017-0645-5

**Published:** 2017-04-04

**Authors:** Anna Banik, Ralf Schwarzer, Izabela Pawlowska, Monika Boberska, Roman Cieslak, Aleksandra Luszczynska

**Affiliations:** 1 CARE-BEH Center for Applied Research on Health Behavior and Health, SWPS University of Social Sciences and Humanities, Wroclaw, Poland; 2 Department of Education and Psychology, Freie Universität Berlin, Berlin, Germany; 3 Institute for Positive Psychology and Education, Australian Catholic University, Strathfield, NSW Australia; 4 Department of Psychology, SWPS University of Social Sciences and Humanities, Warsaw, Poland; 5 Trauma, Health, & Hazards Center, University of Colorado at Colorado Springs, Bluffs Pkwy, Colorado Springs, CO 80918 USA

**Keywords:** Non-small cell lung cancer, Quality of life, Physical symptoms, Self-efficacy, Gender, Family cancer history

## Abstract

**Background:**

We investigated the determinants of trajectories of physical symptoms related to lung cancer (a quality of life [QOL] aspect) and self-efficacy among patients with non-small cell lung cancer (NSCLC). It was hypothesized that gender and family cancer history in first-degree relatives would have synergistic effects on QOL-lung cancer specific symptoms and self-efficacy. Women with family cancer history were expected to be at risk of poorer adjustment.

**Methods:**

Quantitative, longitudinal design was applied. Participants provided their responses at 3–4 days after surgery, 1-month follow-up, and 4-month follow-up. We recruited 102 in-patients (men: 51%) with NSCLC who underwent surgery aimed at removing a lung tumor. Self-report data were collected with QLQ-LC13 and a scale for self-efficacy for managing illness.

**Results:**

Mixed-models analysis indicated that trajectories of physical quality of life (symptoms of lung cancer) as well as self-efficacy were unfavorable among women with family cancer history.

**Conclusions:**

Among NSCLC patients, gender and family cancer history may be considered basic screening criteria for identifying groups of patients at risk for poorer physical ﻿QOL (higher level of physical symptoms related to lung cancer) and lower incline of self-efficacy after cancer surgery.

## Background

Lung cancer is accounting for the largest number of cancer-related deaths in the European Union [1]. Besides poor survival rates [1], lung cancer and lung cancer surgery may cause major changes in some areas of quality of life (QOL). For example, patients with the most frequent form of lung cancer, non-small cell lung cancer (NSCLC), report a decline in physical function across six months after surgery [2, 3]. Lung-cancer specific symptoms such as pain, fatigue, dyspnea, and coughing are reported frequently [3]. Additionally, there are subsets of patients who report persistently poor QOL across months after surgery [3]. Importantly, levels of physical QOL are significantly associated with overall survival: A 10% decline in physical QOL during the first six months after surgery is associated with an 18% increased risk of death [4].

Discovering factors explaining a decline in physical aspects of QOL would help to identify patients who are at risk of poor post-surgery adjustment. Consequently, screening and identifying those at risk for poorer physical functioning would be possible and psychosocial interventions could target those patients with NSCLC who need them most. Our study aims at investigating the potential risk factors that may be easily and early detected, such as family history of cancer and gender, and their effects on changes in QOL and a psychosocial resource variable, self-efficacy.

The family history of cancer is usually defined in the context of immediate family, that is having parents or siblings with cancer [5, 6]. This construct reflects multiple physiological and psychological factors such as heritability, perceived risk for cancer, as well as psychosocial stress referring to providing support/care for a family member with cancer [5–7]. The role of family history of cancer on patients’ functioning was investigated mainly in the context of distress or emotional indices of QOL. High distress levels are particularly likely if individuals with family cancer history cared for a cancer patient or if they lost a family member due to cancer [8]. A combination of having family cancer history and being diagnosed with cancer was the best predictor of symptoms indicating serious mental health risk [7]. Additionally, being diagnosed with lung cancer constitutes a traumatic experience [9].

Although the effects of an interaction between family cancer history and own cancer diagnosis on mental health and distress are well established in the context of cancers of lung or breast [10, 11], the effects of such interaction on physical functioning or physical symptoms were not thoroughly investigated. Researchers indicated that individuals who perceive themselves as at higher risk for cancer due to family cancer history experience a significant increment of distress [10, 11]. Elevated distress levels, in turn, explain poorer physical QOL among cancer patients [12, 13]. Thus, a larger decline in physical aspects of QOL among patients with NSCLC with family cancer history may be expected, compared to patients without family cancer history.

Conservation of resources (COR) theory proposes a dynamic approach to adaptation to stressful events [14, 15], including resource change as a central mechanism. In particular, this theory suggests that being exposed to severe stressors causes a loss or depletion of individual’s resources, such as self-efficacy. Self-efficacy is one of the key personal psychosocial resources operating when individual is exposed to demanding situations [16]. Social cognitive theory [17] suggests that self-efficacy refers to the confidence that one can employ the skills necessary to cope with stress and mobilize one's resources required to meet the situational demands. Self-efficacy determines physical and mental health [17]. Self-efficacy constitutes a source of emotional or social well-being rather than its outcome [17]. People diagnosed with cancer who observe distress and a decline of physical function of their cancer-afflicted family members may harbor pessimistic beliefs about the course of their own cancer [7]. In line with COR [14, 15] it may be assumed that an exposure to stressful events (such as having a family member diagnosed with cancer) would cause a depletion of self-efficacy.

The beneficial role of behavior-specific self-efficacy as a source of QOL or a determinant of health behaviors was shown in studies with mixed samples of cancer patients [18–20], yet its role in adjustment among lung cancer has been rarely investigated. As indicated, lung cancer has its specificity, related to a high decline in physical aspects of QOL/high level of symptoms and low survival rates [1, 2]. Two cross-sectional studies by Porter et al. [21] and Porter, Keefe, Garst, McBride, and Baucom [22] found that self-efficacy referring to symptom and illness management was related to lower physical symptoms and better physical QOL among lung cancer patients. However, a longitudinal investigation showed that pre-surgery self-efficacy for managing illness was unrelated to physical aspect of QOL at 3-month follow-up [23]. Importantly, theories [17] and research [21–23] consider self-efficacy to form a crucial predictor of QOL or other well-being outcomes. Furthermore, trajectories in self-efficacy (such as post-surgery increase or decline over time) represent a meaningful main outcome, which may be considered independently from QOL trajectories [24]. In sum, among lung cancer patients time-lagged associations between self-efficacy and physical QOL are uncertain. The trajectories of these two variables may represent independent outcomes of post-surgery adaptation. Furthermore, the determinants of low levels of self-efficacy or risk factors for low self-efficacy among lung cancer patients are unclear and require further investigation.

The majority of studies testing the effects of gender on QOL among patients with lung cancer showed that men and women do not differ in physical QOL indices (for a review see [3]). Although gender may not affect QOL directly, it may explain differences in QOL between people with and without family history of cancer. Women are more likely than men to uptake a caregiver role if they have a parent or a sibling with cancer (for a review see [25]). Compared to men caregivers, women caregivers are more likely to report higher stress levels and more negative experiences in relation to caregiving, perceive caregiving as an obligation and duty rather than their private choice, spend more hours on caregiving tasks, and consequently report higher burden and lower QOL [25]. Although most of the comparisons were performed for emotional functioning or mental health, gender differences (unfavorable for women caregivers) were also found for the physical aspect of functioning [26]. In sum, an interaction between gender and having family cancer history may determine a decline in personal resources (such as self-efficacy) due to high stress levels as well as lower levels of QOL, also in its physical domain.

We investigated the determinants of trajectories in physical symptoms related to lung cancer (a QOL aspect) and self-efficacy, measured at 3–4 days after surgery, 1-month follow-up, and 4-month follow-up. It was hypothesized that gender and immediate family cancer history would have synergistic effects on symptoms reported by patients with NSCLC. Women with family cancer history were expected to be at risk of poorer physical functioning and poorer self-efficacy.

QOL-lung cancer specific symptoms may be predicted by patient’s emotional well-being and mental health [27]. Therefore, our investigation of the trajectories of QOL-lung cancer specific symptoms will account for potential confounders such as emotional QOL and social QOL. Furthermore, as levels and trajectories of self-efficacy among cancer patients may depend on socio-demographic variables such as age [28] or cancer-related variables (e.g., the type of treatment) [28], these variables will be accounted for in an investigation of self-efficacy trajectories. Although some models discussing the role of self-efficacy in adjustment to cancer would suggest that emotional or social QOL may explain self-efficacy [28], the majority of research and theoretical approaches [17, 18, 21–23] would posit that self-efficacy explains emotional or social QOL instead of assuming that self-efficacy is determined by QOL. Therefore, controlling for baseline emotional and social QOL while explaining self-efficacy trajectories over time may be unnecessary.

## Methods

### Participants

Participants were 102 men (*n* = 52; 51%) and women (*n* = 50; 49%) who underwent surgery aimed at removing a lung tumor, non-small cell lung cancer (NSCLC). All patients provided their responses at Time 1 (T1; 3–4 days after surgery) and Time 2 (T2, 1-month follow-up); two participants died before Time 3 (T3; 4-month follow-up). There was no other attrition. Analyses were conducted in the sample of the completers.

Respondents were 22–79 years old (*M* = 59.31, *SD* = 10.17). The majority of NSCLC patients were married or in a long-term relationship (70%), 15% were widowed, 13% defined themselves as single, and 2% were divorced. The minority had a university degree (20%); 80% had a high school education or lower levels of education.

### Procedures

Participants were recruited among inpatients who received NSCLC-related surgery in the Lower Silesia Center for Lung Diseases, which is the main regional center for lung cancer treatment in South-West Poland. Exclusion criteria were: being hospitalized for other reasons than lung tumor removal surgery, declaring an inability to participate due to difficulties to breathe or speak, staying in the intensive care unit or a high dependency recovery unit for more than 72 h after the surgery. Patients who had a lung surgery aiming at tumor removal were approached and invited to take part in the study, informed about its aims and design. After identifying the potential participants and receiving their consent, the experimenter consulted the ward surgeon about the type of surgery and diagnosis. Only patients with NSCLC who received lobectomy or wedge resection were included. The experimenters were certified clinical psychologists. After each measurement point, all participants were offered psychological consultation. However, no patient made an appointment to take part in such consultations.

All participants provided their informed written consent, and procedures securing respondents’ anonymity were applied. The study was approved by the Internal Review Board at the first author’s institution.

### Measurement of quality of life: lung cancer-related physical symptoms

QLQ-LC13 [29], measuring QOL domain of lung cancer-related symptoms, was used. It includes 12 items referring to lung cancer-associated symptoms (cough, haemoptysis, dyspnea, and site-specific pain), treatment-related side effects (sore mouth, dysphagia, peripheral neuropathy, and alopecia). Although the standard approach to scoring would be to combine three dyspnea items and use the remaining items as nine separate symptom subscales [29], this strategy is not based on a theoretical/empirical approach proposing distinct constructs and it results in multiplying (related) outcomes. Therefore, in the present study we combined the 12 symptoms into one index of QOL referring to physical functioning related to lung cancer. The scale had good reliability with α of .71, .78, and .74 at T1, T2, and T3 respectively.

Scores of the indices obtained with QLQ-LC13 range from 0 to 100. High scores for symptoms scales represent a high level of symptomatology [30]. A difference of 5–10 points in the scores represents a small change, 10–20 points a moderate change and greater than 20 points a large, clinically significant change from the patient’s perspective [31].

### Measurement of self-efficacy for managing illness

This self-efficacy scale was based on a self-efficacy measure used previously in the context of dealing with lung cancer [22]. We used a total of six items from the scale of Porter et al. [22], referring to self-efficacy for managing pain (two items), symptoms (two items), and function (two items). The item example was ‘How certain are you that you can manage your daily activities even if you feel pain due to lung cancer and its treatment?’ or ‘How certain you are that you can do something to help yourself even if you are feeling blue due to your illness and its consequences?’ Additionally, we developed seven items referring to patients’ ability to cope with consequences related to lung cancer-associated symptoms (cough, haemoptysis, dyspnea and site-specific pain), included in QLQ-LC13. For example, patients were asked ‘How certain are you that you can deal with challenges related to your illness and its consequences even if you are short of breath when you walk?’, ‘How certain are you that you can deal with challenges related to your illness and its consequences even if you feel pain in your shoulder/back?’. Six items from the scale by Porter et al. [22] and seven items developed for this study were combined into a single scale, as all items loaded on one factor obtained in a principal component analysis, with factor loadings ranging from .37 to .70. Responses were given on 5-point scale ranging from 1 (definitely not) to 5 (exactly true). Reliability of the scale was good, with Cronbach’s α = .76 at T1, α = .82 at T2, and α = .80 at T3. Modest associations with scores of the General Self-Efficacy Scale [32] were found, with *r* of .30 (T1), .27 (T2), and .31 (T3), all *ps* < .01.

### Measurement of family history of cancer

Patients were asked to indicate if any of their first-degree relatives (a parent, brother, sister, or child) have ever been diagnosed with cancer and treated for cancer. Patients were asked to refer to any type of cancer, occurring any time. Measures using similar formula were applied in previous research (cf. [7]). This general, nominal index with 0–1 values was chosen as it represented the best fit for the hypotheses and analytical strategy investigating if gender and family cancer history differentiate trajectories of QOL-lung cancer symptoms and self-efficacy.

Three out of 102 patients were unsure about family cancer history, but the majority (54%) of the remaining 99 patients reported family cancer history.

### Measurement of covariates

Social and emotional aspects of QOL were measured at T1 with respective subscales of EORTC QLQ-C30 [30]. The responses are given on 5-point scales ranging from 1 (definitely not) to 5 (exactly true) and then recoded to a scale ranging from 0 to 100 [30]. High scores for symptoms scales represent better functioning in respective areas [30]. The subscale assessing emotional QOL includes 4 items (e.g.,’ During the past week did you feel depressed?’). Mean T1 scores were 71.75 (*SD* = 31.49). The subscale assessing social QOL includes 2 items (e.g.,’During the past week has your physical condition or medical treatment interfered with your social activities?’). Mean T1 scores were 79.83 (*SD* = 26.83). The reliability of both measures was acceptable, with alpha of .61 and .51 for emotional and social subscales, respectively.

### Statistical analysis

Using the SPSS 24 MIXED procedure, linear multilevel models were computed with maximum likelihood (ML) estimation [33] using family cancer history and gender as the main predictors. We specified two outcomes (QOL-lung cancer specific physical symptoms and self-efficacy for managing illness) as level-1 dependent variables. Three time points nested in individuals represented level-2 variables. We studied cross-level interactions to determine the interrelationships between family cancer history (coded as: +1 [with family cancer history], −1 [without family cancer history]), gender (coded as −1 [female], +1 [male]), and time (coded as: 0, 1, 2). The analysis explaining the changes in lung cancer-specific QOL accounted for additional emotional QOL and social QOL. The analysis explaining changes in self-efficacy accounted for two covariates, age and surgery type (1 for lobectomy, 0 for segmentectomy). The time-invariant covariates, such as age, emotional QOL, and social QOL were mean-centered.

In a linear mixed-effects model, the responses from participants are thought to be the sum of fixed and random effects. The fixed effects are of primary interest, and random effects contribute to the covariance structure of the data. Adjustments for the covariance structure make the results more accurate. Three patients were unsure whether there has been any cancer diagnosis in their family network. These were defined as missing values across the analyses.

Based on medium-size effects observed in previous research [23] we estimated that the sample securing adequate power to obtain significant effects should include 101 participants. The estimation was done with G*Power 3.1.9.2 software.

Missing data for all variables were replaced using regression (maximum likelihood estimation). In total, 0.07% of the values were replaced.

## Results

### Preliminary analyses

The majority reported that they never observed any lung cancer symptoms (42%) or experienced some symptoms in the year prior the surgery (37%), with remaining 21% of participants indicating severe symptoms. Following tumor stages were reported: 52% of t1 (tumor no larger than 3 cm), 15% of t2 (tumor of 3–7 cm; tumor involves the main bronchus), 3% of t3 (tumor is larger than 7 cm, has grown into the chest wall, the diaphragm, the mediastinal pleura or parietal pericardium) whereas 31% of patients were not certain about the stage of their tumor. The surgery procedures included lobectomy (21%) and wedge resection or segmentectomy (79%). For all patients, it was the first onset of lung cancer; all participants were less than 30 days since the consultation when the suspicion of lung cancer was raised.

We evaluated the effects of stages of cancer and patients’ knowledge about their cancer stage on the study variables. The stages of cancer were unrelated to cancer in family, *χ* ^2^(3, 73) = 3.72, *p* = .249. Patients who knew their cancer stage did not differ in terms of family cancer history from those who were unaware of their cancer stage, *χ* ^2^(1, 100) = 2.80, *p* = .095. Gender was unrelated to patients’ knowledge of their cancer stage, *χ* ^2^(1, 100) = 2.88, *p* = .090. Women and men did not differ in the stages of cancer, *χ* ^2^(3, 73) = 2.30, *p* = .512. The patients who knew the stage of their cancer did not differ from those who did not know the stage in levels of self-efficacy and QOL indicators across the measurement points, all *F*s < 0.46, *p*s > .500. More advanced stages of cancer were related to lower QOL-lung cancer symptoms (i.e., higher symptoms), with *r* = .23 (*p* = .052) at T1, *r* = .31 (*p* = .009) at T2, and *r* = .26 (*p* = .030) at T3. The stages of cancer were unrelated to self-efficacy (*r*s from -.18 to -.06, *p*s > .098).

Correlations between the main study variables, that is QOL (lung cancer-specific symptoms) and self-efficacy indices at three measurement points are reported in Table 1. Between-groups comparisons conducted at three measurement points indicated that gender and family cancer history had negligible or small effects on QOL-lung cancer physical symptoms, but their effects on self-efficacy for managing illness were significant and of small-to-moderate size (Table 1). Cancer in family was unrelated to participant’s gender, *χ* ^2^ (1, 100) = 1.23, *p* = .268.

**Table 1:** Correlations between variables, means and standard deviations (*SD*) of two dependent variables, and pairwise comparisons

		Correlation coefficients					Gender comparisons					Patients with family cancer history vs. those without family cancer history				
	Variable	2	3	4	5	6	Women *M (SD)*	Men *M (SD*)	*F*	*p*	*d* (95% CI)	With *M* (*SD*)	Without *M* (*SD*)	*F*	*p*	*d* (95% CI)
1	QOL-lung cancer symptoms T1	.71***	.66***	−.11	−.17†	−.39***	32.33 (20.73)	26.39 (19.19)	2.41	.124	0.30 (−3.58, 4.17)	33.79 (21.24)	23.73 (17.49)	6.58	.012	0.52 (−3.31, 4.35)
2	QOL-lung cancer symptoms T2		.63***	−.11	−.30**	−.36***	16.33 (14.69)	12.45 (9.65)	2.50	.117	0.32 (−2.10, 2.73)	16.51 (14.21)	11.78 (9.69)	3.68	.058	0.39 (−2.03, 2.80)
3	QOL-lung cancer symptoms T3			−.05	−.30**	−.51***	18.88 (22.58)	9.64 (11.87)	6.32	.014	0.52 (−2.98, 4.02)	18.08 (22.64)	9.90 (10.78)	5.01	.027	0.45 (−3.10, 4.01)
4	Self-efficacy for managing illness T1				.63***	.29**	3.64 (0.59)	4.06 (0.49)	14.56	>.001	−0.78 (−0.89, −0.68)	3.75 (0.61)	3.98 (0.53)	4.01	.048	−0.40 (−0.52, −0.29)
5	Self-efficacy for managing illness T2					.59***	4.38 (0.44)	4.67 (0.29)	15.18	>.001	−0.79 (−0.86, −0.71)	4.49 (0.43)	4.57 (0.37)	1.05	.308	−0.20 (−0.28, −0.12)
6	Self-efficacy for managing illness T3						4.57 (0.36)	4.77 (0.29)	9.07	.003	−0.62 (−0.68, −0.56)	4.61 (0.37)	4.75 (0.30)	4.23	.042	−0.42 (−0.48, −0.35)

For all between-groups comparisons: df = 1,97. *d* = Cohen’s *d*; Significant (CI 95%) effect sizes are reported with Cohen’s *d*

*** *p* < .001; ** *p* < .01; * *p* < .05; † *p* < .10

The effects of gender and family cancer history were observed for QOL-lung cancer symptoms at T1 and self-efficacy at T1 and T3 (Table 1). In particular, women had poorer physical quality of life at T3, as indicated by higher scores of QOL-lung cancer physical symptoms (Cohen’s *d* = 0.52). Participants with family cancer history had poorer physical quality of life at T1 (Cohen’s *d* = 0.52) and T3 (Cohen’s *d* = 0.45). Across all three measurement points, self-efficacy levels were lower for women and participants with family cancer history (Cohen’s ds ranging from 0.20 to 0.79).

### The effects of time, gender, and family cancer history on QOL-lung cancer specific symptoms

First, the analysis aimed at testing the synergistic effects of gender and family cancer history on changes in QOL-lung cancer physical symptoms, measured at 3–4 days after surgery, 1-month follow-up, and 4-month follow-up. Linear mixed models were computed with time points nested in individuals, using QOL-lung cancer physical symptoms at three time points as the level-1 dependent variable, patients family cancer history and gender as level-2 variables, and emotional QOL and social QOL as covariates. Analyses were conducted first without the interaction terms, and then repeated with the interactions between gender, family cancer history and time (Table 2).

**Table 2:** Linear mixed modeling: synergistic effects of time, gender, and family cancer history on QOL-lung cancer specific symptoms

Parameter	Estimate	*SE*	df	*t*	*P*	95% Confidence Intervals	
						Lower Bound	Upper Bound
Results for QOL-lung cancer physical symptoms: analysis with main effects only (no interaction terms)							
Intercept	**26.28**	**1.43**	**162.03**	**18.36**	**<.01**	**23.45**	**29.10**
Emotional QOL (covariate)	**−0.18**	**0.04**	**99.57**	**−4.11**	**<.01**	**−0.27**	**−0.09**
Social QOL (covariate)	**−0.13**	**0.05**	**99.33**	**−2.83**	**.01**	**−0.23**	**−0.04**
Family cancer history	2.22	1.24	99.44	1.79	.08	−0.24	4.68
Gender	−0.62	1.33	99.68	−0.47	.64	−3.27	2.02
Time	**−7.52**	**0.76**	**122.15**	**−9.85**	**<.01**	**−9.03**	**−6.01**
Results for QOL-lung cancer physical symptoms: analysis with interaction terms							
Intercept	**25.96**	**1.40**	**164.12**	**18.60**	**<.01**	**23.21**	**28.72**
Emotional QOL (covariate)	**−0.16**	**0.04**	**99.88**	**−3.68**	**<.01**	**−0.24**	**−0.07**
Social QOL (covariate)	**−0.14**	**0.05**	**99.53**	**−3.03**	**<.01**	**−0.23**	**−0.05**
Family cancer history	**2.97**	**1.41**	**162.46**	**2.10**	**.04**	**0.18**	**5.76**
Gender	0.13	1.49	155.71	0.09	.93	−2.80	3.07
Gender * Family cancer history	−1.94	1.41	162.53	−1.38	.17	−4.73	0.84
Time	**−7.61**	**0.75**	**121.39**	**−10.09**	**<.01**	**−9.10**	**−6.11**
Time * Family cancer history	−0.62	0.75	121.39	−0.82	.41	−2.11	0.88
Time * Gender	−0.74	0.75	121.38	−0.98	.33	−2.23	0.75
Time * Gender* Family cancer history	**−1.32**	**0.75**	**121.38**	**−1.75**	**.08**	**−2.81**	**0.17**

Significant effects (*p* < .05) and trends for significant effects (*p* < .10) are marked in bold

**Fig. 1:**
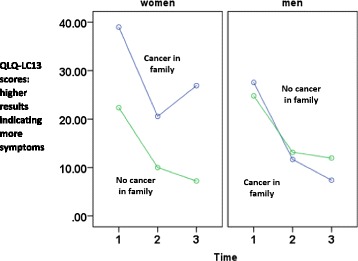
Trajectories of quality of life-lung cancer symptoms (QLQ-LC13 scores, Y axis) across three measurement points (1: 3–4 days after surgery, 2: 1-month follow-up, and 3: 4-month follow-up): Effects of gender and family cancer history among patients with non-small cell lung cancer

The findings are presented in Table 2 and Fig. 1. The intercept of 25.96 indicates the estimated initial status of men with family history of cancer. This analysis was controlled for initial emotional QOL (*b* = −0.16, *t* = −3.68, *p* < .01) and social QOL (*b* = −0.14, *t* = −3.03, *p* < .01), exerting two main effects. Time showed a decreasing overall trend (*b* = −7.61, *t* = −10.09, *p* < .01). Gender (*p* = .93) was not associated with the initial levels of QOL-lung cancer physical symptoms, but family history of cancer was (*b* = 2.97, *t* = 2.10, *p* = .04) which means that patients who have reported having had a family member with cancer also reported higher levels of QOL-lung cancer symptoms. These findings were qualified by a trend for a higher-order interaction of cancer in family * time * gender (*b* = −1.32, *t* = −1.75, *p* = .08) documenting a differential trajectory for men and women across time and between groups with or without cancer in family.

As presented in Fig. 1, lung cancer symptoms declined over time in men with and without family cancer history. A similar, declining trajectory was found among women without family cancer history. However, women with family cancer history reported a high level of lung cancer symptoms at T1, followed by a decline at T2 and an increase of symptoms at T3.

### The effects of time, gender, and family cancer history on self-efficacy

Next, the analysis aimed at testing the synergistic effects of gender and family cancer history on changes in self-efficacy for managing illness, measured at 3–4 days after surgery, 1-month follow-up, and 4-month follow-up. Again, linear mixed models were computed with time points nested in individuals, using self-efficacy at three time points as the level-1 dependent variable, patients’ family history of cancer and gender as level-2 variables, and age and surgery type as covariates. Analyses were conducted first without the interaction terms, and then repeated with the interactions between gender, family cancer history, and time (Table 3).

**Table 3:** Linear mixed modeling: synergistic effects of time, gender, and family cancer history on self-efficacy

Parameter	Estimate	*SE*	df	*t*	*p*	95% Confidence Intervals	
						Lower Bound	Upper Bound
Results for self-efficacy for managing illness: analysis with main effects only (no interaction terms)							
Intercept	**3.89**	**0.05**	**196.43**	**84.88**	**<.01**	**3.80**	**3.98**
Age (covariate)	<−0.01	<0.01	108.64	−1.33	.19	−0.01	<0.01
Surgery type (covariate)	0.11	0.08	109.36	1.47	.14	−0.04	0.27
Family cancer history	**−0.07**	**0.03**	**108.91**	**−2.11**	**.04**	**−0.13**	**<0.01**
Gender	**0.15**	**0.03**	**108.91**	**4.61**	**<.01**	**0.09**	**0.22**
Time	**0.41**	**0.03**	**284.28**	**14.41**	**<.01**	**0.35**	**0.47**
Results for self-efficacy for managing illness: analysis with interaction terms							
Intercept	**3.91**	**0.05**	**469.47**	**86.70**	**<.01**	**3.82**	**3.99**
Age (covariate)	<−0.01	<0.01	689.34	−1.37	.17	−0.01	<0.01
Surgery type (covariate)	0.10	0.08	686.15	1.26	.21	−0.06	0.26
Family cancer history	**−0.08**	**0.04**	**442.34**	**−2.03**	**.04**	**−0.17**	**<0.01**
Gender	**0.21**	**0.04**	**444.84**	**4.92**	**<.01**	**0.12**	**0.29**
Gender * Family cancer history	−0.04	0.04	445.44	−0.85	.40	−0.12	0.05
Time	**0.42**	**0.03**	**259.45**	**15.87**	**<.01**	**0.36**	**0.47**
Time * Family cancer history	0.02	0.03	259.45	0.70	.49	−0.03	0.07
Time * Gender	**−0.06**	**0.03**	**259.45**	**−2.15**	**.03**	**−0.11**	**<−0.01**
Time * Gender* Family cancer history	**0.06**	**0.03**	**259.45**	**2.29**	**.02**	**0.01**	**0.11**

Significant effects (*p* < .05) and trends for significant effects (*p* < .10) are marked in bold

**Fig. 2:**
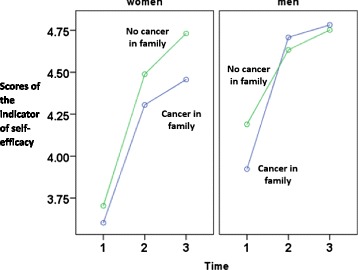
Trajectories of self-efficacy for managing illness (mean item response, Y axis) across three measurement points (1: 3–4 days after surgery, 2: 1-month follow-up, and 3: 4-month follow-up): Effects of gender and family cancer history among patients with non-small cell lung cancer. Self-efficacy mean item responses could range from 1 to 5. As the actual mean item responses were above 4, only values above 4 are displayed in Y axis

The findings on self-efficacy are presented in Table 3 and Fig. 2. The intercept of 3.91 indicates the estimated initial status of men with family history of cancer. This analysis was controlled for initial age (*p* = .17) and surgery type (*p* = .21), both of which did not contribute significantly to the equation. Time showed an increasing overall trend (*b* = 0.42, *t* = 15.87, *p* < .01). Gender was associated with the initial status (*b* = 0.21, *t* = 4.92, *p* < .01), meaning that men had higher self-efficacy. Regarding the linear slope of self-efficacy, men showed a slower rate of change as compared with women (*b* = −0.06, *t* = −2.15, *p* = .03). The family history of cancer was related to self-efficacy (*b* = −0.08, *t* = −2.03, *p* = .04) which means that patients with a family history of cancer reported lower self-efficacy for managing illness. These findings were qualified by the higher-order interaction of cancer in family * time * gender (*b* = 0.06, *t* = 2.29, *p* = .02) which documents a differential trajectory for men and women across time and between groups with and without family cancer history.

As presented in Fig. 2, men with and without family cancer history presented similar self-efficacy changes over time. In particular, self-efficacy levels markedly increased from T1 to T2, and remained high and stable between T2 and T3. The increase of self-efficacy from T1 to T2 was also found for women. However, although among women without family cancer history there was an additional increase from T2 to T3, self-efficacy remained relatively stable from T2 to T3 (and markedly lower) among women with family cancer history.

## Discussion

The findings offer an insight into synergistic effects of NSCLC patients’ gender and family cancer history on a physical aspect of QOL (QOL-lung cancer specific symptoms) and a personal resource variable, self-efficacy for managing illness. A significant time * family cancer history interaction was found for QOL-lung cancer specific symptoms. Furthermore, a trend for a significant interaction of gender, family cancer history and time was found for QOL-lung cancer specific symptoms. Overall, a systematic decline in the level of lung-cancer-specific symptoms was observed among men and women without family cancer history and among men with family cancer history. However, women with family cancer history tended to have had a different trajectory of QOL-lung cancer specific symptoms, with an initial decline followed by an increase of symptoms. Additionally, a significant interaction of gender, family cancer history, and time explained self-efficacy trajectories. A systematic increase of self-efficacy for managing illness was found for men and women without family cancer history and among men with family cancer history. However, the incline of self-efficacy over time was markedly smaller among women with family cancer history. Concluding, among patients with lung cancer surgery, women with family cancer history formed a group that was at-risk for less favorable trajectories of QOL-lung cancer specific symptoms and self-efficacy.

Our study extends the evidence obtained in previous research which indicated effects of being diagnosed with cancer and having family cancer history on mental health outcomes [7, 9]. In particular, we show that synergistic effects of being diagnosed with cancer and having family cancer history may account for the role of the third variable, gender. Furthermore, we show that these synergistic effects are salient for trends in physical aspects of QOL, which are related to mortality among NSCLC patients [4]. Our findings may also help to explain a lack of effects of gender on physical QOL among patients with lung cancer [3]. In line with previous research [3] we found that direct effects of gender on the physical aspect of QOL may be absent or very small. However, gender produced a synergistic effect on physical aspect of QOL, together with family cancer history. In sum, both gender and family cancer history need to be accounted for in explaining QOL among patients with lung cancer.

The underlying mechanisms, explaining the effects of female gender and having an immediate family member diagnosed with cancer, were not investigated in our study. The synergistic effects of these two factors may represent a high likelihood of being an informal caregiver, which in turn may be a proxy of high stress levels and negative experiences in relation to caregiving, longer time spent on caregiving tasks, and burnout-related exhaustion [25, 26]. Another set of underlying mechanisms may refer to cancer in the family (in contrast to other mental or physical health issues). In particular, observing distress and a decline of physical function of family members with cancer may affect patients’ beliefs about their own cancer, its treatment, and prognosis (cf. [5, 7]). Additionally, having an immediate family member with cancer influences one’s own perceived risk for cancer and related distress [10]. Future studies need to clarify the complex mediating mechanisms explaining the synergistic effects of gender and family cancer history.

This study has several limitations. Although three measurement points were applied, they cover a relatively short period after surgery related to NSCLC. Further observations, covering the year after surgery are needed to establish if the observed trends are stable over longer periods. The vast majority of the participants had less advanced stages of NSCLC and they had either lobectomy or wedge resection/segmentectomy. Trajectories observed among patients with the most advanced stages of NSCLC and those with pneumonectomy may be different and no generalizations should be made at this stage. The measurement of family cancer history was very general and referred to any cancer in any immediate family members, partners, or children. Therefore, more detailed analyses investigating the type of cancer, death due to cancer, time of illness and relationship with family members with cancer could not be conducted. As research evidence indicates that depression is a relevant risk factor for poorer physical QOL in lung cancer patients [27] this variable should be carefully controlled in further studies. Unfortunately, our study did not account for a measure of depression. Our study did not account for potentially relevant determinants of self-efficacy or QOL such as social support [34]. Future research may need to further control for potential effects of other social or personal resources. Although our operationalization of family cancer history allows for comparisons with other studies testing the role of this variable [7], it did not allow us for testing the effects of providing care to other family members or members with other chronic and severe illnesses. Future research needs to clarify if the observed effects of family history refer to cancer in the family (and not other illnesses) and to immediate family (versus next of kin, any relative).

## Conclusion

The results point out that female NSCLC patients with family cancer history constitute a group that is at risk for poorer post-surgery adjustment in terms of QOL-lung cancer specific symptoms and selected psychosocial resources, such as self-efficacy. As a decline in physical QOL is related with an increased risk of mortality among patients with lung cancer [4], the findings may have implication for clinical practice. Among people with NSCLC, women with family cancer history should be the primary target for psychosocial interventions. Identifying the at-risk population and targeting these groups constitute the standards of best practices for developing psychosocial interventions [35]. Such interventions may focus on self-efficacy enhancement [20].

Importantly, data referring to gender and self-reported immediate family cancer history are easy to measure and obtain, compared to variables which require complex measurement and interpretation (e.g., psychosocial resource variables). The use of gender and family cancer history as screening criteria should be brief and easy, and therefore, facilitate the screening process for both patients and healthcare professionals.

Regardless of its limitations, our study provides novel evidence and explains trajectories of QOL-lung cancer physical symptoms and self-efficacy among patients with NSCLC. These trajectories were unfavorable among women with family cancer history. Gender and family cancer history may be considered basic screening criteria for identifying groups of patients at risk for poorer physical QOL (higher level of physical symptoms related to lung cancer) and lower incline of self-efficacy after cancer surgery.

## Abbreviations

COR: Conservation of resources theory
ML: Maximum likelihood estimation
NSCLC: Non-small lung cancer
QLQ-LC13: The EORTC study group on quality of life lung cancer-specific questionnaire module
QOL: Quality of life
